# Character of Discharge From the US Military and Suicide Mortality

**DOI:** 10.1001/jamanetworkopen.2025.12081

**Published:** 2025-05-23

**Authors:** Mark A. Reger, Chandru Ravindran, Sybil W. Morley, Andrew Devendorf, Kristen J. Vescera, Brady M. Stephens

**Affiliations:** 1 Veterans Affairs Puget Sound Health Care System, Seattle, Washington; 2 Department of Psychiatry and Behavioral Sciences, University of Washington, Seattle; 3 Veterans Integrated Service Network 2 Center of Excellence for Suicide Prevention, Canandaigua, New York

## Abstract

**Importance:**

Suicide risk may be elevated among individuals who separate from military service with a character of discharge or service that was not honorable (ie, dishonorable, bad conduct, other than honorable, general, or uncharacterized), but a comprehensive evaluation of this possibility has not been previously published.

**Objective:**

To examine suicide mortality by character of discharge compared with individuals who received an honorable discharge and the full veteran population recently separated from service.

**Design, Setting, and Participants:**

This retrospective cohort study included all individuals who separated from the active component of the US military between January 1, 2002, and December 31, 2021. Data were obtained from the US Department of Veterans Affairs/Department of Defense Mortality Data Repository. Suicide rates for each character of discharge were compared with those among individuals receiving an honorable discharge and the full veteran population recently separated from service. Analyses were conducted between December 21, 2023, and June 1, 2024.

**Exposure:**

Character of discharge assigned at separation from the active component of military service.

**Main Outcomes and Measures:**

Crude suicide rates up to 5 years after separation were examined. Age-standardized rate ratios were used for comparisons within the cohort between strata. Age-standardized mortality ratios (SMRs) were used to compare suicide mortality by character of discharge with the full separation cohort and within specific military service branches.

**Results:**

Among 3 627 653 individuals (mean [SD] age at separation, 28.4 [8.6] years; 83% men), 5599 deaths by suicide occurred during the study period. All character of discharge groups had significantly higher suicide rates than the honorable group, with standardized rate ratios ranging from 1.91 (95% CI, 1.69-2.17) for uncharacterized to 2.77 (95% CI, 2.52-3.05) for general. Veterans who received an honorable discharge were less likely to die by suicide than the overall cohort population (SMR, 0.82 [95% CI, 0.79-0.85]), while veterans with all other characters of discharge were more likely to die by suicide, with SMRs ranging from 1.23 (95% CI, 1.15-1.31) for uncharacterized to 1.84 (95% CI, 1.72-1.97) for general. Common demographic risk factors for suicide (eg, age, race) were replicated within character groups in most cases.

**Conclusions and Relevance:**

In this cohort study, individuals who did not receive an honorable character of discharge were at significantly higher risk of suicide compared with veterans who received an honorable discharge and the veteran population recently separated from service. These findings suggest that character of discharge may be a helpful risk factor to consider for ongoing suicide prevention efforts.

## Key Points

**Question:**

What is the association of characters of discharge from US military service with risk of suicide?

**Findings:**

In this cohort study of 3 627 653 individuals, those who received a dishonorable/bad conduct, other than honorable, general, or uncharacterized character of discharge had significantly higher rates of suicide after separation from military service compared with those who had received an honorable character of discharge.

**Meaning:**

These findings support suicide prevention services for individuals who do not receive an honorable discharge from military service.

## Teaser

This cohort study examines suicide mortality rates by character of service discharge among US military veterans.

## Introduction

The age- and sex-adjusted suicide rate for veterans is 71.8% higher than the rate for nonveteran adults.^1^ Risk is not equal for all veterans, however, and there have been substantial concerns that individuals who separate from the military with a character of discharge or service that was not honorable may face unique risks for suicide.^2^ (The term character of discharge is used herein for concision, as not every separation represents a discharge from service.) More than 80% of service members receive an honorable discharge,^3^ which means, in general, that they fulfilled their service to the military and are separating without behavior or performance problems. An honorable discharge provides veterans access to a range of benefits, such as Veterans Health Administration services, US Department of Veterans Affairs (VA) compensation and pension benefits, educational benefits, and home loan benefits from the VA.^4^ Other characters of discharge are considered either administrative (general under honorable conditions [general], other than honorable [OTH]) or punitive (bad conduct, dishonorable).^4^ General or OTH discharges occur in instances such as less-than-satisfactory performance or a pattern of misconduct. Bad conduct and dishonorable discharges often occur as a result of felony-level offenses and are determined by a court-martial.^5^ Dishonorable is the most punitive discharge, and the determination is often associated with a prison sentence. An additional administrative discharge called uncharacterized is often assigned to individuals who served less than 180 days in the military.^6^ The laws and policies that define the character of discharge and associated benefits are complex, but in general, individuals with less-than-honorable characters of discharge receive fewer benefits.^2^

Character of discharge may be an important indicator of suicide risk for several reasons. Research has highlighted associations among a variety of adverse outcomes and a character of discharge that was less than honorable. Compared with honorable and general discharges, individuals with an OTH or a punitive discharge are more likely to report physical and mental health problems, such as substance use, depression, and suicidal ideation.^7,8^ In a small study, self-reported suicide attempts were not associated with character of discharge, but risk of homelessness was more than 4 times as high among a combined group of veterans with either an OTH or a punitive character of discharge compared with those with an honorable character of discharge.^9^ Individuals with an OTH or a punitive discharge are also more likely to be single, have less social support, and have a history of incarceration.^10^ A bad conduct or dishonorable discharge may contribute to difficulties in obtaining employment and receiving governmental assistance,^11,12^ including federal grants to pursue higher education.^13^ It is also possible that other preenlistment characteristics or experiences during military service may differ from the general veteran population in some ways that confer suicide risk.

Despite the intense interest in this topic,^2^ we are not aware of a peer-reviewed examination of suicide risk by type of character of discharge. To our knowledge, only 1 preliminary analysis examined suicide mortality and character of discharge. Separated service members who received any character of discharge besides honorable (a combined variable that included general, bad conduct, OTH, and dishonorable) had a higher hazard of suicide than those who received an honorable character of discharge.^14^ Since this analysis mixed administrative and punitive characters of discharge that vary substantially in severity, a more detailed analysis is needed. In addition, it would be helpful to know whether risk varies by demographic or military characteristics within each type of character of discharge.^1^

To inform suicide prevention efforts, this evaluation examined whether demographic characteristics were associated with suicide rates within 5 years of separation for different characters of discharge. We also evaluated whether some characters were associated with a higher risk of suicide compared with (1) individuals who separated with an honorable discharge and (2) the broader veteran population from which they were drawn. We examined whether these patterns differed by military service branch since suicide risk differs by branch among recently separated veterans.^15^

## Methods

### Study Population

This retrospective, population-based cohort study included individuals who separated from the active component of the US military (Air Force, Army, Marine Corps, Navy) between January 1, 2002, and December 31, 2021. The methods and data ascertainment for analyses were considered non–human participant research and did not require institutional review board approval per the VA Office of Research and Development *Program Guide 1200.21*,^16^ and informed consent was not required. This study followed the Strengthening the Reporting of Observational Studies in Epidemiology (STROBE) reporting guideline.

Separation was defined as either a discharge from the active component into civilian status or a transition from the active component into the reserve component. For service members with multiple transitions, we used the data associated with their most recent separation. Data related to service characteristics and demographics, including race and ethnicity, were obtained from the VA/Department of Defense Identity Repository (VADIR) and were examined because they have been associated with suicide outcomes in prior studies.^17^ VADIR is an electronic repository of military personnel’s military history, their payroll information, and their dependents’ data.^18^ Marital status and education are refreshed within VADIR if the veteran updates their information, so the values assigned for this study reflect either data from the time of separation or the most recent postseparation value at the time of analysis. Race data included American Indian or Alaska Native; Asian, Native Hawaiian, or Other Pacific Islander (combined to permit analyses); Black or African American (hereafter referred to as Black); and White. Ethnicity was categorized as Hispanic or not Hispanic.

### Exposure Variables and Suicide Ascertainment

Character of discharge assigned at separation from the active component was the primary exposure variable obtained from VADIR, which receives the character of discharge field along with military personnel data from the Department of Defense. If an individual had more than 1 separation, the discharge character of the latest separation was used based on the character recorded in VADIR. One individual whose last separation was honorable for VA purposes was excluded from the cohort. Dishonorable–dismissal and bad conduct were combined into 1 category due to small cell sizes (hereafter referred to as dishonorable/bad conduct). Other than honorable is an official administrative category that represents 1 type of character of discharge^4^ and is not a category created by the research team to group other characters of discharge.

Service members entered the cohort on their separation date and were followed up for 5 years (1825 days) or until their date of death or the end of the follow-up period (December 31, 2021), whichever came first. We examined the first 5 years after separation because we wanted to focus on service members who recently transitioned out of military service. Suicide and all-cause mortality data were obtained from the VA/Department of Defense Mortality Data Repository, which compiles data from annual VA and Department of Defense searches of death certificate data from the National Death Index.^19^ *International Statistical Classification of Diseases, Tenth Revision*^20^ codes X60 to X84, Y87.0, and U03 were used to identify deaths by suicide.

### Statistical Analysis

Crude suicide rates within 5 years after separation were calculated. Directly age-standardized rate ratios (SRRs) and 95% modified confidence intervals based on the γ-distribution, as developed by Tiwari et al,^21^ were generated for comparisons within the cohort between strata. Age-standardized mortality ratios (SMRs) and exact 95% CIs based on a Poisson distribution were used to compare suicide mortality by character of discharge with the full separation cohort and within specific military service branch. Records missing character of discharge (5.8%) were excluded from analysis. Otherwise, missing data were handled via pairwise deletion. We also performed sensitivity analyses of male-stratified results given the small cell sizes for female veterans. These findings are provided in eTables 1 to 7 in Supplement 1. Analyses were conducted from December 21, 2023, through June 1, 2024, using SAS Enterprise Guide, version 8.3 (SAS Institute Inc). The threshold for significance was set at a 2-sided *P* < .05.

## Results

The study cohort included 3 627 653 individuals (mean [SD] age at separation, 28.4 [8.6] years; 83% men and 17% women). Two percent of cohort individuals were of American Indian or Alaska Native race, and 6% were of Asian, Native Hawaiian, or Other Pacific Islander; 17% of Black; and 70% of White race. Eleven percent of cohort individuals were of Hispanic and 88% of non-Hispanic ethnicity. There were 5599 deaths by suicide within 5 years of separation.

### Demographic Characteristics by Character of Discharge

Table 1 displays the crude suicide rates and SRRs within each character group and for each demographic characteristic. Results are described for groups that had sufficient data to present (Table 1; Table 2) as the National Death Index requires the suppression of cell sizes of less than 10 due to privacy concerns.

**Table 1.** Association of Suicide and Demographic Characteristics by Character of Discharge Among Service Members Who Separated From 2002 to 2021

Character of discharge and demographic characteristic	Suicide deaths, No.	Crude suicide rate, per 100 000	SRR (95% CI)^a^
**Honorable**			
Sex			
Female	197	9.97	1 [Reference]
Male	2890	29.53	3.06 (2.65-3.54)^b^
Age at separation, y^c^			
17-22	813	39.01	1.84 (1.68-2.02)^b^
23-27	1143	26.37	1.25 (1.15-1.35)^b^
≥28	1131	21.17	1 [Reference]
Race			
American Indian or Alaska Native	65	34.32	1.21 (0.94-1.56)
Asian, Native Hawaiian, or Other Pacific Islander	225	30.80	1.15 (1.00-1.32)^b^
Black or African American	332	17.73	0.67 (0.60-0.76)^b^
White	2343	28.07	1 [Reference]
Ethnicity			
Hispanic	268	20.89	0.75 (0.66-0.85)^b^
Not Hispanic	2810	26.98	1 [Reference]
Education level at separation			
Non–high school graduate	444	37.52	1.40 (1.26-1.55)^b^
High school graduate	2421	27.50	1 [Reference]
Higher degree	217	12.58	0.43 (0.32-0.59)^b^
Marital status at separation			
Never married or single	2266	30.36	1.39 (1.25-1.55)^b^
Married	707	18.84	1 [Reference]
Divorced, separated, or widowed	79	22.75	1.15 (0.83-1.60)
Branch			
Air Force	535	21.30	0.84 (0.76-0.93)^b^
Army	1300	27.88	1 [Reference]
Marine Corps	752	35.08	1.17 (1.06-1.29)^b^
Navy	500	20.46	0.75 (0.68-0.84)^b^
**General**			
Sex			
Female	33	28.61	1 [Reference]
Male	825	86.79	2.99 (2.11-4.25)^b^
Age at separation, y^c^			
17-22	498	86.22	1.18 (0.95-1.47)
23-27	264	73.93	1.01 (0.80-1.28)
≥28	96	73.16	1 [Reference]
Race			
American Indian or Alaska Native	25	114.77	1.30 (0.87-1.94)
Asian, Native Hawaiian, or Other Pacific Islander	96	93.40	1.05 (0.85-1.31)
Black or African American	154	56.91	0.65 (0.54-0.77)^b^
White	552	88.89	1 [Reference]
Ethnicity			
Hispanic	64	56.56	0.68 (0.53-0.88)^b^
Not Hispanic	786	83.62	1 [Reference]
Education level at separation			
Non–high school graduate	172	92.51	1.18 (1.00-1.39)^b^
High school graduate	672	78.58	1 [Reference]
Higher degree	12	57.67	0.28 (0.10-0.81)^b^
Marital status at separation			
Never married or single	739	81.59	0.96 (0.75-1.24)
Married	94	72.42	1 [Reference]
Divorced, separated, or widowed	11	101.76	2.09 (0.82-5.33)
Branch			
Air Force	94	49.70	0.56 (0.45-0.70)^b^
Army	504	86.97	1 [Reference]
Marine Corps	118	130.29	1.45 (1.18-1.77)^b^
Navy	142	68.68	0.79 (0.66-0.96)^b^
**Other than honorable**			
Sex			
Female	14	32.49	1 [Reference]
Male	412	75.93	2.37 (1.39-4.04)^b^
Age at separation, y^c^			
17-22	261	76.04	1.20 (0.88-1.64)
23-27	118	70.11	1.11 (0.79-1.55)
≥28	47	63.35	1 [Reference]
Race			
American Indian or Alaska Native	NR	NR	NR
Asian, Native Hawaiian, or Other Pacific Islander	38	87.10	1.13 (0.80-1.59)
Black or African American	65	51.17	0.65 (0.49-0.85)^b^
White	296	80.13	1 [Reference]
Ethnicity			
Hispanic	44	64.32	0.86 (0.63-1.18)
Not Hispanic	382	74.68	1 [Reference]
Education level at separation			
Non–high school graduate	82	76.06	1.07 (0.84-1.36)
High school graduate	333	71.29	1 [Reference]
Higher degree	10	126.57	3.03 (1.31-7.03)^b^
Marital status at separation			
Never married or single	389	76.46	1.45 (0.89-2.35)
Married	28	44.04	1 [Reference]
Divorced, separated, or widowed	NR	NR	NR
Branch			
Air Force	13	88.83	1.23 (0.66-2.29)
Army	122	71.08	1 [Reference]
Marine Corps	190	103.77	1.40 (1.11-1.77)^b^
Navy	101	46.67	0.64 (0.49-0.83)^b^
**Dishonorable/bad conduct**			
Sex			
Female	NR	NR	1 [Reference]
Male	93	67.44	NR
Age at separation, y^c^			
17-22	27	68.94	1.39 (0.77-2.50)
23-27	47	69.86	1.41 (0.83-2.40)
≥28	19	49.59	1 [Reference]
Race			
American Indian or Alaska Native	NR	NR	NR
Asian, Native Hawaiian, or Other Pacific Islander	NR	NR	NR
Black or African American	12	35.45	0.49 (0.27-0.91)^b^
White	69	72.90	1 [Reference]
Ethnicity			
Hispanic	NR	NR	NR
Not Hispanic	86	67.64	1 [Reference]
Education level at separation			
Non–high school graduate	15	63.19	0.97 (0.56-1.68)
High school graduate	78	65.68	1 [Reference]
Higher degree	NR	NR	NR
Marital status at separation			
Never married or single	76	65.66	0.83 (0.44-1.56)
Married	16	63.55	1 [Reference]
Divorced, separated, or widowed	NR	NR	NR
Branch			
Air Force	11	46.69	0.71 (0.35-1.41)
Army	32	66.18	1 [Reference]
Marine Corps	40	75.81	1.12 (0.69-1.82)
Navy	10	49.80	0.74 (0.36-1.52)
**Uncharacterized**			
Sex			
Female	82	22.27	1 [Reference]
Male	824	74.45	3.32 (2.64-4.17)^b^
Age at separation^c^			
17-22	740	63.21	1.59 (1.13-2.23)^b^
23-27	131	60.54	1.52 (1.05-2.21)^b^
≥28	35	39.80	1 [Reference]
Race			
American Indian or Alaska Native	25	89.68	1.31 (0.88-1.95)
Asian, Native Hawaiian, or Other Pacific Islander	83	66.75	0.97 (0.77-1.22)
Black or African American	84	35.17	0.52 (0.42-0.66)^b^
White	684	67.75	1 [Reference]
Ethnicity			
Hispanic	49	35.04	0.54 (0.40-0.72)^b^
Not Hispanic	857	64.98	1 [Reference]
Education level at separation			
Non–high school graduate	173	84.67	1.50 (1.27-1.77)^b^
High school graduate	699	58.03	1 [Reference]
Higher degree	14	37.12	0.52 (0.11-2.33)
Marital status at separation			
Never married or single	818	64.77	1.72 (1.23-2.41)^b^
Married	56	35.61	1 [Reference]
Divorced, separated, or widowed	NR	NR	NR
Branch			
Air Force	104	54.20	0.90 (0.73-1.12)
Army	407	59.33	1 [Reference]
Marine Corps	206	90.19	1.46 (1.22-1.74)^b^
Navy	189	51.25	0.86 (0.72-1.02)

Abbreviations: NR, not reported (data suppressed due to small cell size); SRR, standardized rate ratio.

^a^ Variables adjusted for age.

^b^ Significant at *P* < .05.

^c^ Unadjusted rate ratio was used when age was the variable of interest.

**Table 2.** Comparison of Suicide Rates by Character of Discharge Among Service Members Who Separated From 2002 to 2021

Character	Suicide deaths, No.	Crude suicide rate, per 100 000	SRR (95% CI)^a^
**Overall**			
Honorable	3087	26.25	1 [Reference]
General	858	80.49	2.77 (2.52-3.05)^b^
Dishonorable/bad conduct	93	64.25	2.22 (1.79-2.75)^b^
Other than honorable	426	72.72	2.49 (2.18-2.83)^b^
Uncharacterized	906	61.42	1.91 (1.69-2.17)^b^
**Air Force**			
Honorable	535	21.30	1 [Reference]
General	94	49.70	1.91 (1.26-2.89)^b^
Dishonorable/bad conduct	11	46.69	1.79 (0.92-3.47)
Other than honorable	13	88.83	3.68 (1.96-6.93)^b^
Uncharacterized	104	54.20	1.22 (0.87-1.73)
**Army**			
Honorable	1300	27.88	1 [Reference]
General	504	86.97	2.95 (2.60-3.33)^b^
Dishonorable/bad conduct	32	66.18	2.08 (1.44-3.00)^b^
Other than honorable	122	71.08	2.25 (1.82-2.78)^b^
Uncharacterized	407	59.33	1.74 (1.49-2.03)^b^
**Marine Corps**			
Honorable	752	35.08	1 [Reference]
General	118	130.29	3.24 (2.60-4.03)^b^
Dishonorable/bad conduct	40	75.81	2.11 (1.52-2.91)^b^
Other than honorable	190	103.77	2.76 (2.28-3.34)^b^
Uncharacterized	206	90.19	2.18 (1.59-2.99)^b^
**Navy**			
Honorable	500	20.46	1 [Reference]
General	142	68.68	2.84 (2.24-3.60)^b^
Dishonorable/bad conduct	10	49.80	2.22 (1.16-4.26)^b^
Other than honorable	101	46.67	2.42 (1.81-3.22)^b^
Uncharacterized	189	51.25	2.75 (1.96-3.85)^b^

Abbreviation: SRR, standardized rate ratio.

^a^ Adjusted for age.

^b^ Significant at *P* < .05.

While effect sizes differed somewhat by character of discharge, the general demographic patterns were similar. Men had significantly higher rates of suicide than women for every character group that had sufficient data to analyze with SRRs, which ranged between 2.37 (95% CI, 1.39-4.04) for OTH and 3.32 (95% CI, 2.64-4.17) for uncharacterized. Black individuals had significantly lower suicide rates than White individuals for all character groups, with SRRs ranging from 0.49 (95% CI, 0.27-0.91) for dishonorable/bad conduct to 0.67 (95% CI 0.60-0.76) for honorable. Asian, Native Hawaiian, or Other Pacific Islander individuals had higher rates than White individuals for the honorable group (SRR, 1.15 [95% CI, 1.00-1.32]). Hispanic individuals had lower rates than non-Hispanic individuals for every character of discharge group that had sufficient data to analyze, with SSRs ranging from 0.54 (95% CI, 0.40-0.72) for uncharacterized to 0.75 (95% CI 0.66-0.85) for honorable; OTH had no significant difference.

Differences were noted by service branch, with the Army as the referent (Table 1). In the honorable and general groups, suicide rates were lower in the Air Force (SRR, 0.84 [95% CI, 0.76-0.93] and 0.56 [95% CI, 0.45-0.70], respectively) and Navy (SRR, 0.75 [95% CI, 0.68-0.84] and 0.79 [95% CI, 0.66-0.96], respectively), while rates were higher for the Marine Corps (SRR, 1.17 [95% CI, 1.06-1.29] and 1.45 [95% CI, 1.18-1.77], respectively). In the OTH group, the same pattern was observed for the Marine Corps and Navy (SRR, 1.40 [95% CI, 1.11-1.77] and 0.64 [95% CI, 0.49-0.83], respectively); no significant difference was found between the Air Force and Army. The Marine Corps also had higher rates than the Army in the uncharacterized group (SRR, 1.46 [95% CI, 1.22-1.74]). No differences were observed by service branch in the dishonorable/bad conduct group. Results for other demographic characteristics (eg, education, marital status) are presented in Table 1.

### Comparisons With Honorable Character of Discharge

All character of discharge groups had significantly higher suicide rates than the honorable group (Table 2). The SRRs were greater than 2.0 in most cases and ranged from 1.91 (95% CI, 1.69-2.17) for uncharacterized to 2.77 (95% CI, 2.52-3.05) for general. When service branch was considered, this pattern of elevated rates compared with honorable character of discharge was consistent across service branches, with 2 exceptions: Dishonorable/bad conduct and uncharacterized in the Air Force showed no differences compared with honorable.

### Comparisons With All Recently Separated Veterans

The honorable group had a lower risk of suicide within 5 years of separation (SMR, 0.82 [95% CI, 0.79-0.85]) compared with all recently separated veterans, while all other character of discharge groups had a higher risk of suicide, with SMRs ranging from 1.23 (95% CI, 1.15-1.31) for uncharacterized to 1.84 (95% CI, 1.72-1.97) for general (Figure). When results were examined by service branch, this pattern remained consistent for all branches except for Air Force and Navy in the dishonorable/bad conduct group and Air Force in the uncharacterized group, which showed no differences compared with all recently separated veterans.

**Figure.**
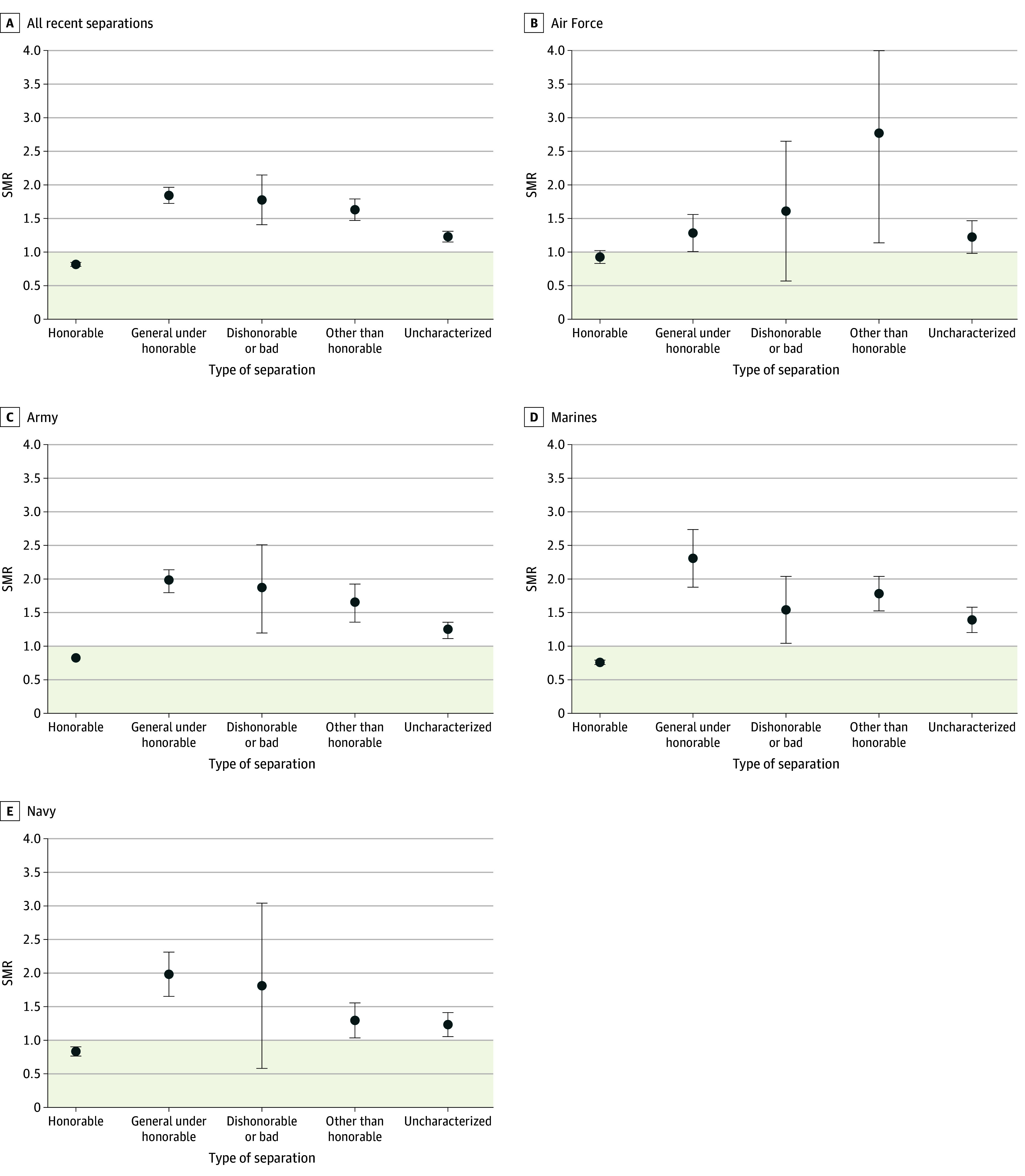
Standardized Mortality Ratios (SMRs) for Recently Separated Veterans With Character of Discharge by Branch Data are standardized for age, with the population of service members who separated from 2002 to 2021 as the standard. Standardized mortality ratios greater than 1.0 indicate an increased risk of mortality, while SMRs less than 1.0 indicate a reduced risk of mortality. Error bars represent 95% CIs.

## Discussion

This cohort study is the first in our knowledge to comprehensively evaluate suicide risk by character of discharge or service among individuals in the 5 years following separation from the military. The results show that individuals who received any administrative or punitive character of discharge had a significantly elevated risk of suicide compared with those who received an honorable character of discharge. The magnitude of the effect sizes was notable as most character of discharge groups showed rates that were more than twice the suicide rate of the honorable group after standardizing by age. The general, OTH, dishonorable/bad conduct, and uncharacterized groups also had significantly higher risks of suicide than the recently separated veteran population.

The data analyzed here do not provide reasons for these associations. Some research has suggested that individuals in some of the more punitive character of discharge groups experience elevated rates of unemployment, homelessness, and relationship problems, which are also suicide risk factors.^10,22^ However, it was interesting that the magnitude of the associations we observed did not vary according to the severity of the character of discharge. For example, all the groups had high SRRs compared with the honorable character of discharge group; the groups that received administrative characters of discharge, such as general and OTH, had elevated rates similar to the dishonorable/bad conduct groups. This pattern of results is counterintuitive since the individuals in the dishonorable group often are convicted of a felony-level charge and serve time in prison.^10,23,24^ We expected this group to have the highest suicide rates. We speculate that all these characters of discharge may be associated with suicide risk factors such as impulsivity, despair, or stress. It is possible that the individuals in these groups had preenlistment characteristics or experiences that conferred risk or experiences during or after military service that increased risk. For example, individuals who did not receive an honorable character of discharge may have had higher rates of mental health problems.^3,9,10^ Future studies should control for these factors. However, since mental health problems often go undetected among individuals at risk for suicide (and some of these individuals may not qualify for Veterans Health Administration services that could detect mental health disorders), character of discharge may still serve as a helpful risk factor for suicide prevention purposes. It is also possible that psychological and emotional effects of administrative and punitive characters of discharge may vary due to personal interpretation (eg, shame and guilt vs feeling mistreated or a sense of injustice or institutional betrayal), regardless of the type of discharge.

The results also suggest that the demographic suicide risk factors that have been identified in prior veteran (and civilian) studies were replicated in this cohort across most character of discharge groups. For example, White, non-Hispanic men had some of the highest suicide rates compared with their respective demographic comparison groups in most or all cases, regardless of character of discharge group. Demographic risk factors were similar to the overall veteran population.^1^

Executive orders,^25^ congressional legislation,^26^ and VA policies^2^ have all worked to expand mental health and suicide prevention resources in recent years to some groups that did not receive an honorable discharge. The current data cannot be used to evaluate the success of those efforts, but the data support the importance of suicide prevention services among individuals who separate from the military with a character of discharge that was not honorable. Formal program evaluations could be developed to estimate the impact of specific policy changes.

### Limitations

This study has several limitations. The evaluation period included data from 2002 to 2021, and policies managing character of discharge and benefits have changed, especially for OTH. For example, effective June 25, 2024, a new rule expanded VA benefits to some veterans with a dishonorable character of discharge.^2^ Additionally, our analysis was limited to separations from the active component, and results for the reserve component may differ. The military service branches may also differ in how they assign discharge type. We recognize that the character of discharge an individual receives is an indication of documented behavior and may not reflect actual behavior conducted during their service. In addition, since all individuals studied were within 5 years of separation, we did not examine how our results may differ by time since separation. Future studies should examine whether suicide risk changes over time (eg, 10 or 20 years after separation). We could not adjust analyses by sex due to small cell sizes for female veterans, so we provided male-stratified results in our sensitivity analyses to confirm that results were not influenced by inclusion of women.

## Conclusions

In this cohort study of more than 3.6 million individuals who separated from military service, those who did not receive an honorable character of discharge were at a significantly higher risk of suicide compared with veterans who received an honorable discharge and the recently separated veteran population. These findings suggest that character of discharge may be a helpful risk factor to consider for ongoing suicide prevention efforts.

## Notes

Supplement 1. **eTable 1. **Standardized Rate Ratios by Demographic Characteristics for Male Service Members Who Separated With an Honorable Character of Discharge, 2002-2021 **eTable 2.** Standardized Rate Ratios by Demographic Characteristics for Male Service Members Who Separated With a General Character of Discharge, 2002-2021 **eTable 3.** Standardized Rate Ratios by Demographic Characteristics for Male Service Members Who Separated With an Other Than Honorable Character of Discharge, 2002-2021 **eTable 4.** Standardized Rate Ratios by Demographic Characteristics for Male Service Members Who Separated With a Dishonorable/Bad Conduct Character of Discharge, 2002-2021 **eTable 5.** Standardized Rate Ratios by Demographic Characteristics for Male Service Members Who Separated With an Uncharacterized Character of Discharge, 2002-2021 **eTable 6.** Standardized Rate Ratios by Character of Discharge Among Male Service Members Who Separated From 2002 to 2021, by Branch of Service **eTable 7.** Standardized Mortality Ratios by Character of Discharge Among Male Service Members Who Separated From 2002 to 2021

Supplement 2.
Data Sharing Statement
