# Supplementary material for: Character of Discharge From the US Military and Suicide Mortality
Source: JAMA Netw Open. 2025 May 23;8(5):e2512081. doi: 10.1001/jamanetworkopen.2025.12081 (PMC12102701; doi:10.1001/jamanetworkopen.2025.12081)
Supplement: Supplement 1. — eTable 1. Standardized Rate Ratios by Demographic Characteristics for Male Service Members Who Separated With an Honorable Character of Discharge, 2002-2021 eTable 2. Standardized Rate Ratios by Demographic Characteristics for Male Service Members Who Separated With a General Character of Discharge, 2002-2021 eTable 3. Standardized Rate Ratios by Demographic Characteristics for Male Service Members Who Separated With an Other Than Honorable Character of Discharge, 2002-2021 eTable 4. Standardized Rate Ratios by Demographic Characteristics for Male Service Members Who Separated With a Dishonorable/Bad Conduct Character of Discharge, 2002-2021 eTable 5. Standardized Rate Ratios by Demographic Characteristics for Male Service Members Who Separated With an Uncharacterized Character of Discharge, 2002-2021 eTable 6. Standardized Rate Ratios by Character of Discharge Among Male Service Members Who Separated From 2002 to 2021, by Branch of Service eTable 7. Standardized Mortality Ratios by Character of Discharge Among Male Service Members Who Separated From 2002 to 2021 [file jamanetwopen-e2512081-s001.pdf]

Supplemental Online Content

Reger MA, Ravindran C, Morley SW, Devendorf A, Vescera KJ, Stephens BM. Character of discharge from the US military and suicide mortality. *JAMA Netw Open*. 2025;8(5):e2512081.
doi:10.1001/jamanetworkopen.2025.12081

eTable 1. Standardized Rate Ratios by Demographic Characteristics for Male Service Members Who Separated With an Honorable Character of Discharge, 2002-2021

eTable 2. Standardized Rate Ratios by Demographic Characteristics for Male Service Members Who Separated With a General Character of Discharge, 2002-2021

eTable 3. Standardized Rate Ratios by Demographic Characteristics for Male Service Members Who Separated With an Other Than Honorable Character of Discharge, 2002-2021

eTable 4. Standardized Rate Ratios by Demographic Characteristics for Male Service Members Who Separated With a Dishonorable/Bad Conduct Character of Discharge, 2002-2021

eTable 5. Standardized Rate Ratios by Demographic Characteristics for Male Service Members Who Separated With an Uncharacterized Character of Discharge, 2002-2021

eTable 6. Standardized Rate Ratios by Character of Discharge Among Male Service Members Who Separated From 2002 to 2021, by Branch of Service

eTable 7. Standardized Mortality Ratios by Character of Discharge Among Male Service Members Who Separated From 2002 to 2021

This supplemental material has been provided by the authors to give readers additional information about their work.

© 2025 Reger MA et al. *JAMA Network Open*.

Objective:

This appendix contains male stratified analyses as a supplement to main analysis. Cell sizes were not sufficiently sized to adjust analyses by sex, so this supplement is provided to show that results do not differ from main analysis substantially when looking at males only.

© 2025 Reger MA et al. *JAMA Network Open*.

eTable 1. Standardized Rate Ratios by Demographic Characteristics for Male Service Members Who Separated With an Honorable Character of Discharge, 2002-2021

Category	Level	Unadjusted Rate Per 100,000	SRR	SRR 95% LCL	SRR 95% UCL
Age Group*	Age 17-22	45.73	**1.95**	**1.78**	**2.15**
	Age 23-27	29.88	**1.28**	**1.17**	**1.39**
	Age 28+	23.40	REF	REF	REF
Race	American Indian/Alaskan Native	41.08	**1.31**	**1.01**	**1.70**
	Asian/Native Hawaiian/Other Pacific Islander	32.70	1.12	0.97	1.30
	Black/African American	21.85	**0.77**	**0.68**	**0.87**
	White	30.82	REF	REF	REF
Ethnicity	Hispanic	22.90	**0.73**	**0.64**	**0.84**
	Not Hispanic	30.35	REF	REF	REF
Branch	Air Force	24.97	0.90	0.81	1.01
	Army	31.30	REF	REF	REF
	Marines	37.06	1.09	0.99	1.20
	Navy	23.06	**0.77**	**0.69**	**0.85**
Education Level	Did Not Graduate High School	41.65	**1.39**	**1.25**	**1.54**
	High School Graduate	30.84	REF	REF	REF
	Higher Degree	14.20	**0.41**	**0.31**	**0.55**
Marital Status	Never Married or Single	34.39	**1.39**	**1.24**	**1.56**
	Married	20.60	REF	REF	REF
	Divorced/Separated/Widowed	30.24	1.34	0.94	1.91

Note: Bold font indicates p<0.05; SRR=standardized rate ratio, LCL=Lower Confidence Limit, UCL=Upper Confidence Limit

*Unadjusted Rate Ratios were calculated for age groups. All other RRs are adjusted by age.

© 2025 Reger MA et al. *JAMA Network Open*.

eTable 2. Standardized Rate Ratios by Demographic Characteristics for Male Service Members Who Separated With a General Character of Discharge, 2002-2021

Category	Level	Unadjusted Rate Per 100,000	SRR	SRR 95% LCL	SRR 95% UCL
Age Group*	Age 17-22	94.56	1.21	0.97	1.51
	Age 23-27	77.84	1.00	0.79	1.27
	Age 28+	78.03	REF	REF	REF
Race	American Indian/Alaskan Native	116.77	1.24	0.81	1.89
	Asian/Native Hawaiian/Other Pacific Islander	95.54	1.01	0.81	1.26
	Black/African American	63.68	**0.68**	**0.57**	**0.82**
	White	94.77	REF	REF	REF
Ethnicity	Hispanic	61.58	**0.69**	**0.54**	**0.90**
	Not Hispanic	89.82	REF	REF	REF
Branch	Air Force	52.74	**0.56**	**0.44**	**0.71**
	Army	92.36	REF	REF	REF
	Marines	141.63	**1.48**	**1.21**	**1.82**
	Navy	76.70	0.84	0.69	1.01
Education Level	Did Not Graduate High School	97.92	1.16	0.97	1.37
	High School Graduate	84.89	REF	REF	REF
	Higher Degree	64.69	**0.31**	**0.10**	**0.97**
Marital Status	Never Married or Single	87.52	0.93	0.72	1.20
	Married	79.74	REF	REF	REF
	Divorced/Separated/Widowed	115.82	1.88	0.65	5.44

Note: Bold font indicates p<0.05; SRR=standardized rate ratio, LCL=Lower Confidence Limit, UCL=Upper Confidence Limit
*Unadjusted Rate Ratios were calculated for age groups. All other RRs are adjusted by age.

© 2025 Reger MA et al. *JAMA Network Open*.

eTable 3. Standardized Rate Ratios by Demographic Characteristics for Male Service Members who Separated with an Other than Honorable Character of Discharge, 2002-2021

Category	Level	Unadjusted Rate Per 100,000	SRR	SRR 95% LCL	SRR 95% UCL
Age Group*	Age 17-22	79.27	1.17	0.86	1.60
	Age 23-27	72.76	1.07	0.76	1.51
	Age 28+	67.83	REF	REF	REF
Race	White	82.76	REF	REF	REF
	Asian/Native Hawaiian/Other Pacific Islander	91.56	1.15	0.81	1.63
	Black/African American	53.92	**0.66**	**0.50**	**0.86**
	American Indian/Alaskan Native	Suppressed	Suppressed	Suppressed	Suppressed
Ethnicity	Hispanic	66.29	0.85	0.62	1.17
	Not Hispanic	77.85	REF	REF	REF
Branch	Air Force	90.34	1.15	0.60	2.20
	Army	74.53	REF	REF	REF
	Marines	106.86	**1.38**	**1.08**	**1.75**
	Navy	48.71	**0.64**	**0.49**	**0.84**
Education Level	Did Not Graduate High School	79.94	1.08	0.84	1.37
	High School Graduate	74.42	REF	REF	REF
	Higher Degree	**125.82**	**2.74**	**1.08**	**6.95**
Marital Status	Never Married or Single	79.42	1.42	0.86	2.35
	Married	47.10	REF	REF	REF
	Divorced/Separated/Widowed	Suppressed	Suppressed	Suppressed	Suppressed

Note: Bold font indicates p<0.05; SRR=standardized rate ratio, LCL=Lower Confidence Limit, UCL=Upper Confidence Limit
"Suppressed" means less than 10 deaths overall in this group
*Unadjusted Rate Ratios were calculated for age groups. All other RRs are adjusted by age.

© 2025 Reger MA et al. *JAMA Network Open*.

eTable 4. Standardized Rate Ratios by Demographic Characteristics for Male Service Members Who Separated With a Dishonorable/Bad Conduct Character of Discharge, 2002-2021

Category	Level	Unadjusted Rate Per 100,000	SRR	SRR 95% LCL	SRR 95% UCL
Age Group*	Age 17-22	72.78	1.40	0.78	2.52
	Age 23-27	73.20	1.41	0.83	2.40
	Age 28+	51.93	REF	REF	REF
Race	White	75.82	REF	REF	REF
	Asian/Native Hawaiian/Other Pacific Islander	Suppressed	Suppressed	Suppressed	Suppressed
	Black/African American	38.17	**0.51**	**0.27**	**0.94**
	American Indian/Alaskan Native	Suppressed	Suppressed	Suppressed	Suppressed
Ethnicity	Hispanic	Suppressed	Suppressed	Suppressed	Suppressed
	Not Hispanic	71.06	REF	REF	REF
Branch	Air Force	51.54	0.75	0.37	1.50
	Army	69.55	REF	REF	REF
	Marines	77.61	1.09	0.67	1.77
	Navy	52.61	0.75	0.36	1.53
Education Level	Did Not Graduate High School	65.73	0.96	0.55	1.66
	High School Graduate	69.02	REF	REF	REF
	Higher Degree	Suppressed	Suppressed	Suppressed	Suppressed
Marital Status	Never Married or Single	68.82	0.82	0.44	1.54
	Married	66.78	REF	REF	REF
	Divorced/Separated/Widowed	Suppressed	Suppressed	Suppressed	Suppressed

Note: Bold font indicates $p<0.05$; SRR=standardized rate ratio, LCL=Lower Confidence Limit, UCL=Upper Confidence Limit
"Suppressed" means less than 10 deaths overall in this group.
*Unadjusted Rate Ratios were calculated for age groups. All other RRs are adjusted by age.

© 2025 Reger MA et al. *JAMA Network Open*.

eTable 5. Standardized Rate Ratios by Demographic Characteristics for Male Service Members Who Separated With Uncharacterized Character of Discharge, 2002-2021

**Category**	**Level**	**Unadjusted Rate Per 100,000**	**SRR**	**SRR 95% LCL**	**SRR 95% UCL**
Age Group*	Age 17-22	77.46	**1.79**	**1.23**	**2.59**
	Age 23-27	71.20	**1.64**	**1.09**	**2.46**
	Age 28+	43.37	REF	REF	REF
Race	White	80.10	REF	REF	REF
	Asian/Native Hawaiian/Other Pacific Islander	72.80	0.91	0.71	1.17
	Black/African American	47.16	**0.60**	**0.47**	**0.76**
	American Indian/Alaskan Native	105.76	1.30	0.84	2.00
Ethnicity	Hispanic	43.79	**0.56**	**0.41**	**0.76**
	Not Hispanic	77.84	REF	REF	REF
Branch	Air Force	67.29	0.92	0.73	1.16
	Army	71.68	REF	REF	REF
	Marines	103.63	**1.36**	**1.14**	**1.63**
	Navy	62.14	0.85	0.71	1.03
Education Level	Did Not Graduate High School	99.42	**1.45**	**1.22**	**1.72**
	High School Graduate	70.58	REF	REF	REF
	Higher Degree	48.95	0.63	0.13	2.93
Marital Status	Never Married or Single	78.15	**1.59**	**1.11**	**2.28**
	Married	43.50	REF	REF	REF
	Divorced/Separated/Widowed	Suppressed	Suppressed	Suppressed	Suppressed

Note: Bold font indicates p<0.05; SRR=standardized rate ratio, LCL=Lower Confidence Limit, UCL=Upper Confidence Limit
"Suppressed" means less than 10 deaths overall in this group.
*Unadjusted Rate Ratios were calculated for age groups. All other RRs are adjusted by age.

© 2025 Reger MA et al. *JAMA Network Open*.

eTable 6. Standardized Rate Ratios by Character of Discharge Among Male Service Members Who Separated From 2002 to 2021, by Branch of Service

Character	Suicides (n)	Suicide Rate Per 100,000	SRR[a] (95% Confidence Limits)
**Overall**			
Honorable	2,890	29.53	REF
General	825	86.79	**2.62 (2.37, 2.89)**
Other than Honorable	412	75.93	**2.30 (2.02, 2.63)**
Bad Conduct/ Dishonorable	93	67.44	**2.05 (1.65, 2.55)**
Uncharacterized	824	74.45	**1.96 (1.72, 2.24)**
**Air Force**			
Honorable	488	24.97	REF
General	87	52.74	**1.73 (1.10, 2.71)**
Other than Honorable	12	90.34	**3.23 (1.66, 6.30)**
Bad Conduct/ Dishonorable	11	51.54	1.63 (0.83, 3.19)
Uncharacterized	92	67.29	1.20 (0.84, 1.73)
**Army**			
Honorable	1,209	31.30	REF
General	485	92.36	**2.74 (2.41, 3.12)**
Other than Honorable	115	74.53	**2.11 (1.69, 2.62)**
Bad Conduct/ Dishonorable	32	69.55	**1.94 (1.35, 2.80)**
Uncharacterized	366	71.68	**1.77 (1.49, 2.09)**
**Marine Corps**			
Honorable	735	37.06	REF
General	116	141.63	**3.32 (2.66, 4.14)**
Other than Honorable	188	106.86	**2.68 (2.21, 3.25)**
Bad Conduct/ Dishonorable	40	77.61	**2.04 (1.47, 2.81)**
Uncharacterized	201	103.63	**2.36 (1.71, 3.24)**
**Navy**			
Honorable	458	23.06	REF
General	137	76.70	**2.73 (2.14, 3.49)**
Other than Honorable	97	48.71	**2.21 (1.64, 2.99)**
Bad Conduct/ Dishonorable	10	52.61	**2.04 (1.06, 3.93)**
Uncharacterized	165	62.14	**2.78 (1.93, 4.00)**

[a] Adjusted for age; Bold font indicates $p<0.05$
SRR=standardized rate ratio; suicide rates are crude rates

© 2025 Reger MA et al. *JAMA Network Open*.

eTable 7. Standardized Mortality Ratios by Character of Discharge among Male Service Members who Separated From 2002 to 2021

Character	SMR[a] (95% Confidence Limits)
**Overall**	
Honorable	**0.82 (0.79, 0.85)**
General	**1.73 (1.62, 1.86)**
Other than Honorable	**1.48 (1.34, 1.63)**
Bad Conduct/ Dishonorable	**1.64 (1.32, 2.01)**
Uncharacterized	**1.29 (1.20, 1.38)**
**Air Force**	
Honorable	0.93 (0.85, 1.02)
General	1.12 (0.90, 1.38)
Other than Honorable	**2.33 (1.20, 4.07)**
Bad Conduct/ Dishonorable	1.49 (0.74, 2.66)
Uncharacterized	1.24 (1.00, 1.52)
**Army**	
Honorable	**0.79 (0.75, 0.84)**
General	**1.79 (1.64, 1.96)**
Other than Honorable	**1.48 (1.22, 1.77)**
Bad Conduct/ Dishonorable	**1.71 (1.17, 2.41)**
Uncharacterized	**1.25 (1.13, 1.39)**
**Marine Corps**	
Honorable	**0.77 (0.71, 0.82)**
General	**2.35 (1.94, 2.82)**
Other than Honorable	**1.70 (1.47, 1.97)**
Bad Conduct/ Dishonorable	**1.48 (1.06, 2.01)**
Uncharacterized	**1.49 (1.29, 1.71)**
**Navy**	
Honorable	**0.84 (0.77, 0.92)**
General	**1.90 (1.59, 2.24)**
Other than Honorable	1.15 (0.93, 1.40)
Bad Conduct/ Dishonorable	1.66 (0.80, 3.05)
Uncharacterized	**1.26 (1.07, 1.47)**

Note: [a] Standardized for age with the population of service members who separated from 2002-2021 as the standard; Bold font indicates p<0.05; Suicides (n) and suicide rates are available in Table S6

© 2025 Reger MA et al. *JAMA Network Open*.
