# Supplementary material for: Character of Discharge From the US Military and Suicide Mortality
Source: JAMA Netw Open. 2025 May 23;8(5):e2512081. doi: 10.1001/jamanetworkopen.2025.12081 (PMC12102701; doi:10.1001/jamanetworkopen.2025.12081)
Supplement: Supplement 2. — Data Sharing Statement [file jamanetwopen-e2512081-s002.pdf]

Data Sharing Statement

Reger. Character of Discharge From the US Military and Suicide Mortality. *JAMA Netw Open*. Published May 23, 2025. doi:10.1001/jamanetworkopen.2025.12081

Data
Data available: No

Additional Information
Explanation for why data not available: Data sharing may be possible through the creation of a de-identified data set and the development of a data use agreement with VA privacy officers that complies with all applicable laws and regulations.
